# A millennium of trophic stability in Atlantic cod (*Gadus morhua*): transition to a lower and converging trophic niche in modern times

**DOI:** 10.1038/s41598-021-92243-7

**Published:** 2021-06-16

**Authors:** Guðbjörg Ásta Ólafsdóttir, Ragnar Edvardsson, Sandra Timsic, Ramona Harrison, William P. Patterson

**Affiliations:** 1 Research Centre of the Westfjords, University of Iceland, Hafnargata 9b, IS415 Bolungarvík, Iceland; 2 Saskatchewan Isotope Laboratory, University of Saskatchewan, 114 Science Place, Saskatoon, SK S7N 5E2 Canada; 3 Department of Archaeology, History, Cultural Studies and Religion, University of Bergen, Øysteinsgate 3, 5007 Bergen, Norway

**Keywords:** Biodiversity, Stable isotope analysis

## Abstract

Stable isotope analyses of zooarchaeological material can be used to examine ecological variability in exploited species at centennial to millennial scales. Climate change is a notable driver of marine ecosystem change, although historical fishing is also likely to have impacted past marine systems. Fishing removes the oldest and largest individuals and may thereby result in shorter trophic pathways and reduced niche width of predatory fish species. In the current study we examine the trophic niche of Atlantic cod, haddock and Atlantic wolffish, in the last millennium using δ^13^C and δ^15^N values of bone collagen. We report a lower trophic level of Atlantic cod and haddock but higher level of wolffish in present times, following centuries at consistent and higher trophic levels of Atlantic cod. This results in a concurrent converging trophic niche of the demersal fish. We suggest that the current data set provides a valuable historical baseline facilitating interpretation of current variability in the trophic ecology of northern demersal fish.

## Introduction

Under the current scenario of rapidly changing marine environments, management decisions may benefit from long term ecological baselines^1–6^. Bio-chemical measures of zooarchaeological material can, for example, be used to approximate ecological traits at a centennial or millennial scale for an estimation of the level of change in the ecosystem before intensifying human exploitation^7–11^. Generally, long term uninterrupted archaeological data series are rare. Commercialization of Atlantic cod (*Gadus morhua*) fishing starting in the Late Medieval Period^12^ and historical fishing sites in Iceland show consistent occupation until the modernization of the fisheries in the late nineteenth century^13^. The quantity and high temporal resolution of zooarchaeological material at these sites make Atlantic cod a useful model to retrospectively examine marine ecological shifts.

δ^15^N values are used as a proxy of trophic position and, in combination with δ^13^C values, allow to characterize the niche of marine species^14^. At the base of the food chain, δ^13^C values reflect the rate of primary production and are highly correlated to ocean temperature and dissolved CO_2_, but also reflect variation in primary producers, for example, inshore vs. offshore, or pelagic vs. benthic habitats^15,16^. δ^15^N values display a stepwise increase from prey to predator and thereby correlate with the consumer’s trophic level^14,17^. Stable isotope analyses of historical and pre-historical specimens have been successfully used to reconstruct historical marine trophic dynamics in relation to population change and demographics^18^, resolve migratory history^19^ and document niche shifts^20,21^.

Climate change and fisheries are the most notable drivers of current marine ecosystem change. The ecological effects of climate change are multifaceted, including ocean acidification and invasive non-native species, but change in ocean temperature alone can have severe effects on distribution and changes in the feeding migration of marine fish^22,23^. Changes in the distribution and migration of Atlantic cod around Iceland are well known from the twentieth and twenty-first centuries but may also have occurred much earlier^24^. Cooling of the Northern Hemisphere started in the early fourteenth century, often associated with a temperature minimum, “the Little Ice Age”, in the seventeenth century, followed by a colder more variable climate^25–29^. Previous research on Atlantic cod zooarchaeological material showed slower growth of juveniles and loss of genetic variation at this time^30–32^. Although the current level of ocean warming is unprecedented, examining marine trophic dynamics across the last millennium of climate fluctuations may reveal patterns of stability or plasticity that in turn may offer scenarios of future effects.

Historical fishing is likely to have impacted marine environments well before modern times, for example, by reducing population sizes and mean age of fish stocks^33,34^. The impacts of fishing on fish trophic dynamics can be complex. “Fishing down the food web”^35^, commonly results in shorter trophic pathways and reduced niche width of predatory fish species as the largest fish and species are removed from the ecosystem^36^. The resulting competitive release can also result in converging trophic levels as more species can utilize favored prey^37^. Conversely, exploitation has also been found to result in longer, less overlapping food webs, likely also related to the removal of apex predators that reduce trophic competition^38,39^. Long term data series of marine trophic ecology reconstructed from zooarchaeological samples may be particularly useful in documenting effects of fishing on trophic ecology in pristine ecosystems.

The main objective of the current study was to determine the long term historical trophic niche of Atlantic cod as well as parallels in the trophic niche of Atlantic cod and other common North Atlantic demersal fish species, that is, haddock (*Melanogrammus aeglefinus*) and Atlantic wolffish (*Anarhichas lupus*). We used δ^13^C and δ^15^N values from zooarchaeological samples dated to Common Era (CE) 890-CE 1910 and present-day bones from the same species to examine variation in species trophic niche, niche width and niche overlap. Specifically, we expected that periods of variable ocean temperatures would coincide with parallel shifts in carbon isotope variability, reflecting change in ocean carbon isotope values and primary productivity. Moreover, we expected fisheries impacts, following the onset of modern fisheries in the late nineteenth century, to be signaled by a lower trophic level and a narrower niche of the highly targeted Atlantic cod as well as converging niches of the three species as stock densities decrease. We do not attempt to reconstruct the diet of the demersal fish as this largely reflects baseline isotope values^40^. Including only samples from north western Iceland minimizes geographical variation in isotope values and strengthen any inferences based on temporal changes (that almost certainly include variation in ocean baseline values) and comparison between species.

## Material and methods

### Sampling and samples

**Figure 1:**
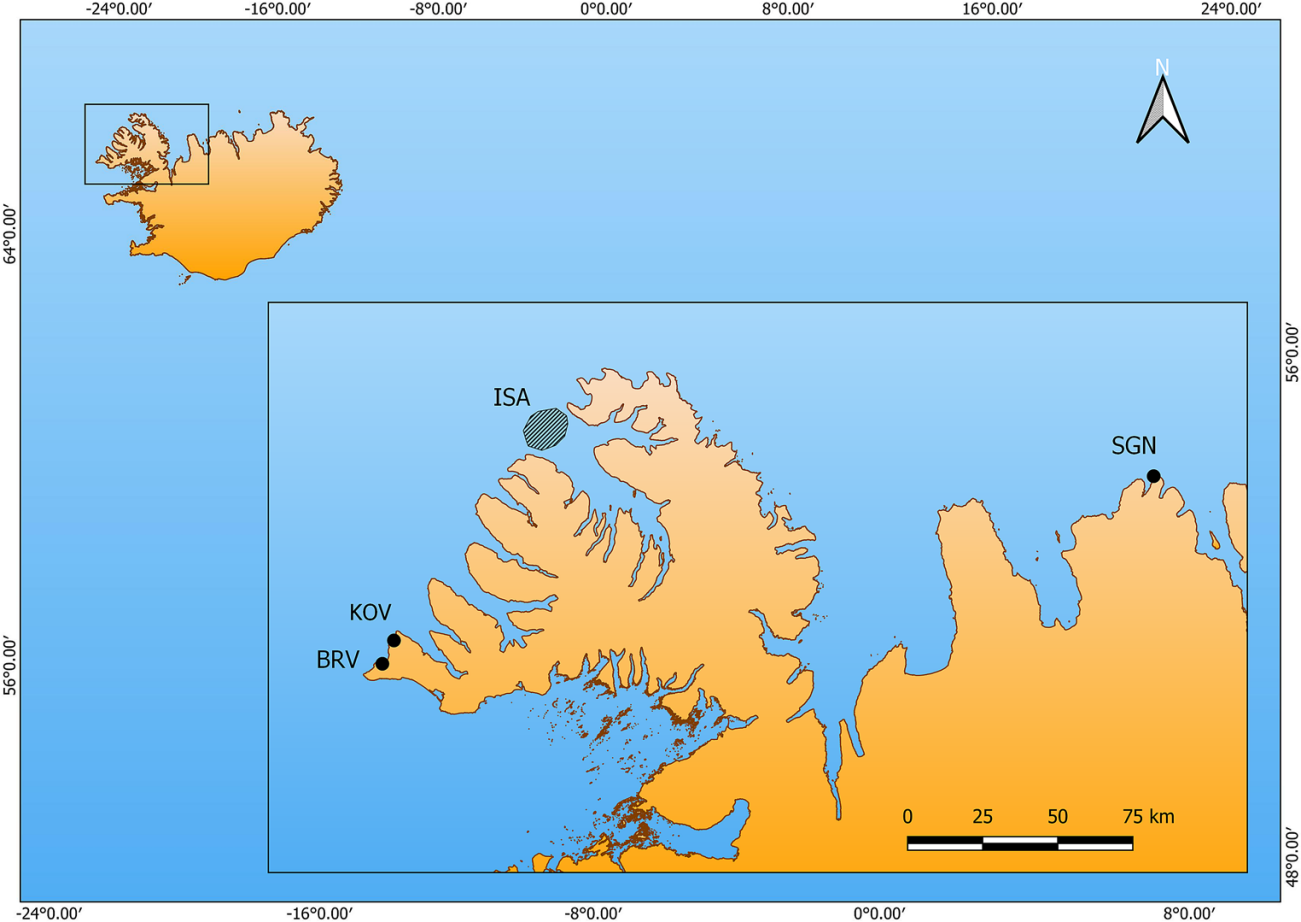
Map showing the excavation sites of Breiðavík (BRV), Kollsvík (KOV) and Siglunes (SGN) as well as the approximate position of the fishing grounds of the present-day comparison samples (ISA) indicated as a shaded area.

Archaeological excavations were carried out at three historical fishing sites in Iceland in the years of 2012, 2015, 2017 and 2019 (Fig. 1). Trenches were excavated into midden deposits exposed by coastal erosion, employing the *single context* method, that is, each individual archaeological deposit was excavated and recorded separately. All contexts, that is, each archaeological deposit that could be uniquely separated from other deposits, were numbered, cleaned, photographed, planned, and finally removed. This allowed maximum temporal resolution of the samples, but many contexts were later dated to the same or similar times (Supplementary Table S1). The minimum size of each trench was 1 × 1 m. All excavated contexts were dry-sieved with a 4 mm mesh and all finds were removed and analyzed. Fish bones were removed from the bone assemblage and analyzed separately.

From the excavations of Breiðavík (BRV) and Kollsvík (KOV), in NW Iceland and Siglunes (SGN) in N Iceland (Fig. 1) a subset of the Atlantic cod vertebrae was used for stable isotope analysis. Haddock vertebrae and wolffish dentary bone (the only wolffish skeleton elements found well preserved) from Breiðavík were used for analysis. This is unlikely to affect comparisons as Bas and Cardona^41^ found no differences in the stable isotope ratios of different skeletal elements in fish species with acellular bone. The Atlantic cod thoracic vertebrae were chosen when possible to minimize the risk of sampling the same individual more than once but for haddock this was not possible because of the much lower bone availability. However, articulated bone elements were never sampled. For wolffish a maximum of two dentary elements could originate from the same individual. As an additional precaution, identical stable isotope values from the same species and context were never used in analysis. Bones were identified to species by morphology and only samples that were unambiguously identified were used for analysis. The archaeological excavations were permitted by the Icelandic Cultural Heritage Agency: Permits no: 21505-0060 (NW Iceland), 201106-0020 (N Iceland).

Identical bone elements were obtained from the modern fishery, that is, vertebrae samples from Atlantic cod and haddock and dentary samples from wolffish. The fish were caught in March 2018 and April 2021 by longline fishing boats targeting fishing grounds in NW Iceland (Fig. 1). The samples were obtained from the fishing company, Jakob Valgeir ehf, using commercial fishing quota allocated from the Icelandic Ministry of Fisheries.

Archaeological material was dated based on the archaeological stratigraphic sequence and ^14^C dates of each archaeological layer. For statistical analysis we pooled samples from archaeological contexts dated to the same century. Terrestrial herbivore bones, when present, were used to date the deposits by ^14^C dating (Scottish Universities Environmental Research Centre). Two archaeological layers from BRV had no ^14^C information available, the dates of these groups were estimated based on finds and stratigraphic sequence information. Specifically, these were a layer dated to CE 1637-1680, assigned the seventeenth century time group and a layer dated to 1410-1649, assigned a sixteenth century time group. The samples originating in northern Iceland were pooled with the samples from NW Iceland as the isotope values did not differ significantly. One of two archaeological layers from SGN was dated by ^14^C to 1239 ± 25 while a second layer was dated by ^14^C to CE 1350 ± 50^42,43^, as there were only three bones dated to the thirteenth century both layers were assigned to a fourteenth century time group. Summary information on the samples analyzed is presented in Table 1 and the full data and dating information is presented in Supplementary Table S1.

**Table 1:** Summary information of the zooarchaeological material used for analysis as well as stable isotope ellipse area estimates, SEA_B_, including 95% confidence intervals (CI) of the SEA_B_ mode.

Century	Atlantic cod							Wolffish							Haddock						
	n	δ^13^C		δ^15^N		SEA_B_		n	δ^13^C		δ^15^N		SEA_B_		n	δ^13^C		δ^15^N		SEA_B_	
		Mean	SD	Mean	SD	Mode	95% CI		Mean	SD	Mean	SD	Mode	95% CI		Mean	SD	Mean	SD	Mode	95% CI
10th	19	− 13.54	1.59	13.45	0.63	3	1.87–4.84	–	–	–	–	–	–	–	2	− 12.05	1.06	12.75	1.2	–	–
14th	25	− 12.42	0.89	13.11	0.64	1.76	1.17–2.61	–	–	–	–	–	–	–	2	− 12.25	0.07	12.8	0	–	–
16th	17	− 12.22	0.88	13.29	0.64	1.47	0.87–2.44	6	− 12.93	0.46	11.95	0.7	0.4	0.17–1.06	5	− 14.72	2.84	13.36	1.55	8	2.28–23.18
17th	19	− 14.1	2.02	13.31	0.51	2.96	1.91–4.92	7	− 12.41	0.78	12.1	0.92	0.72	0.32–1.76	–	–	–	–	–	–	–
18th	12	− 12.38	0.88	13.16	0.57	1.52	0.77–2.83	6	− 14.35	0.78	12.45	0.21	3.06	1.30–8.31	1	− 15.2	NA	13.1	NA	–	–
19th	10	− 13.21	1.26	12.45	1.35	4.42	2.40–9.26	12	− 14.71	0.67	12.03	0.22	0.85	0.48–1.59	1	− 13.4	NA	12.4	NA	–	–
21st	10	− 15.11	0.62	12.6	0.41	0.74	0.38–1.51	16	13.48	1.04	12.13	0.43	1.07	0.63–1.82	7	− 14.89	0.36	12.23	0.48	0.47	0.20–1.10

### Stable isotope analysis

Loose sediment and soil were removed from the bones by brush, while fine cleaning was performed in an ultrasonic bath (model 550D, VWR), with samples immersed in deionized water for 4 min. Samples were oven-dried overnight at 60 °C. Dry samples were powdered in a swing mill. Lipid was extracted from all the present-day samples and or archaeological samples, humic acid was removed prior to analysis. Collagen was extracted according to Leyden et al.^44^. Stable isotope values were obtained using a Thermo Finnigan Flash 1112 EA coupled to a Thermo Finnigan Delta Plus XL via a Conflo III interface. Carbon isotope ratios were corrected for ^17^O contribution using the Craig correction^45^ and reported in per mil notation relative to the Vienna PeeDee Belemnite scale (VPDB). Nitrogen isotope values are reported in per mil notation relative to the Atmospheric Air Reference Scale (AIR). Carbon data were calibrated against the international standards L-SVEC (δ^13^C =  − 46.6‰_VPDB_) and IAEA-CH6 (δ^13^C =  − 10.45‰_VPDB_). Nitrogen data were calibrated against the international standards USGS-25 (δ^15^N =  − 30.4‰_AIR_) and IAEA-305A (δ^15^N = 39.8‰_AIR_). Standard deviation of standards was < 0.01‰ across all batches.

Samples were evaluated for collagen preservation by confirming that their atomic C/N ratio fell within the range of 2.9 and 3.6^46^. δ^13^C values from the present-day bones were corrected for changes in oceanic dissolved inorganic carbon (the Suess Effect), using Eq. (1) defined by Hilton et al.^47^ as quoted in Szpak et al.^48^:

1 $$\Delta ^{{13}} C_{{Suess}} = \alpha _{{Water{\text{~}}body}} \times e^{{\left( {years{\text{~}}from{\text{~}}1850} \right){\text{b}}}}$$

where the shape of the exponential curve is defined by b, the global decrease in oceanic δ^13^C established as 0.027 by Gruber et al.^49^. We approximated the annual rate of decrease, α, as − 0.015‰, based on previous estimates for similar latitudes^50,51^. This resulted in an addition of 1.29‰ to the present-day δ^13^C values.

### Statistical analysis

Examination of residuals from a linear model examining variation in δ^13^C and δ^15^N values across time demonstrates that a linear parametric model does not adequately fit the data. Therefore, we first used a non-parametric Kruskal–Wallis test and a post-hoc pairwise Wilcoxon test to determine if δ^13^C and δ^15^N values differed between centuries for each species.

**Figure 2:**
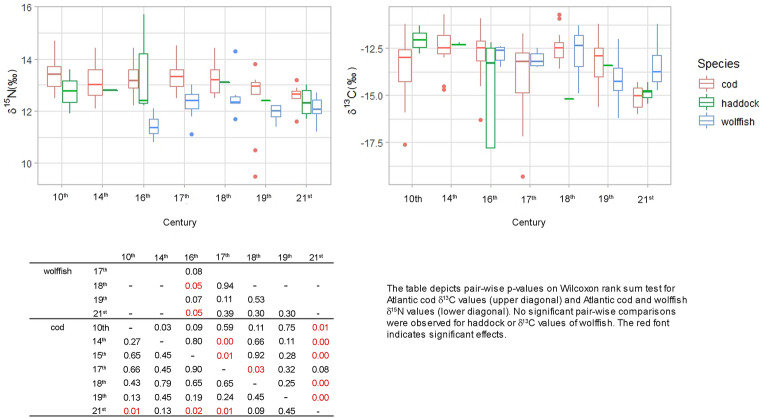
δ^13^C and δ^15^N values from all of the samples used dated from 970 CE and until present-day. The box plots show the stable isotope values in each of the temporal groups, specifically, the median (horizontal line), first (box) and third (whiskers) quartile. Values falling out with the third quartile are presented as circles.

To illustrate non-linear patterns over time and to test whether the variation in carbon and nitrogen isotope values followed a similar curve through time for all fish species (as may for example have been expected by visual examination of the carbon values presented in Fig. 2) we compared two generalized additive models (GAMs) for each isotope. Examination of the residuals from the GAMs supported the use of the models for the current data. GAMs allow a continuous estimation of isotope variability across the centuries with a 95% confidence interval. The GAMs were modelled with a Gaussian distribution, an identity link function, a cubic regression spline and a smoothing parameter on time (k = 7). The first model fitted the same curve for all species.

2 $${\text{E}}\left( {{\text{y}}_{{\text{i}}} } \right) = \beta _{0} + {\text{f}}\left( {{\text{x}}_{{\text{i}}} } \right)$$

whereas the second models allowed different curves between species.

3 $${\text{E}}\left( {{\text{y}}_{{\text{i}}} } \right) = \beta _{0} + {\text{f}}\left( {{\text{x}}_{{\text{i}}} } \right){\text{z}}_{{\text{i}}}$$

where f is the smooth function of isotope values across time and z_i_ represents species. AIC values were used to choose the better model for both carbon and nitrogen isotope values. The models were fitted with the package mgcv in R and validated by the residual simulation and examination implemented in the R package DHARMa^52^.

Isotopic niches of each temporal group and species were calculated and visualized using the SIBER (Stable Isotope Bayesian Ellipses in R) version 2.0 package^53^ for all groups with a sample size of 5 or more δ^13^C and δ^15^N values. This resulted in exclusion of haddock except in the sixteenth century sample and present times. The standard ellipse area (SEA) is a bivariate equivalent of standard deviation and represents the “core isotopic niche”^54^. SEA_B_ is the ellipse area estimated using Bayesian modelling. The Bayesian model uses a Gibbs sampler to fit multivariate normal distributions around each group and was run using the default priors and 10^6^ iterations. By estimating the distribution further inferences can be drawn, such as, a statistical comparison of the ellipse size of time groups by calculating the proportion of the SEA_B_ ellipse estimates that differed in size^53^. SEA_B_ was calculated, and ellipse size compared between centuries. We then determined the pairwise niche overlap between species where they co-existed using the R package nicheROVER^55^ to calculate the probability of finding group A in the niche region of group B.

Finally, δ^15^N values of fish bone collagen may be expected to increase with the age and size of individual fish, as fish often become more piscivorous and forage on larger prey as they grow. Therefore, we examined the relationship between the number of annual rings and δ^15^N values using a GLM. The vertebrae growth rings could only be counted for a subset of the samples.

## Results

All samples included in the analysis had C:N ratios between 2.9 and 3.6, indicative of well-preserved collagen (Table 1). δ^13^C values ranged from − 19.3 to − 10.7‰ and δ^15^N values from 8.2 to 15.7‰. Stable isotope values from 108 Atlantic cod, 31 wolffish and 11 haddock archaeological samples as well as 10 Atlantic cod vertebrae, 7 haddock vertebrae and 16 wolffish jaws, from fish caught in 2018 and 2021, were used for analysis (Table 1; Fig. 2).

The Kruscal–Wallis test showed that δ^15^N values of Atlantic cod vertebrae differed between centuries (χ^2^ = 16.683, df = 6, p value = 0.010) as did the δ^15^N values of wolffish χ^2^ = 13.751, df = 4, p value = 0.008). Pairwise comparisons showed that the values of present-day Atlantic cod differed significantly from all but the values of eighteenth and nineteenth century cod. Pairwise comparison of wolffish δ^15^N values showed that the sixteenth century values were lower than those in the eighteenth century and in present times. Pairwise p values can be found embedded in Fig. 2. The δ^13^C values of Atlantic cod vertebrae differed between centuries (χ^2^ = 34.779, df = 6, p < 0.001) as did the δ^13^C values of wolffish jaws (χ^2^ = 9.072, df = 4, p = 0.05). Pairwise comparisons showed that the Atlantic cod values were generally lower in the seventeenth century and in the present-day samples (Fig. 2). No pairwise comparisons of δ^13^C values were significant for wolffish. No significant differences of stable isotope values between centuries were found for haddock, but the sample size was very low (Table 1).

**Figure 3:**
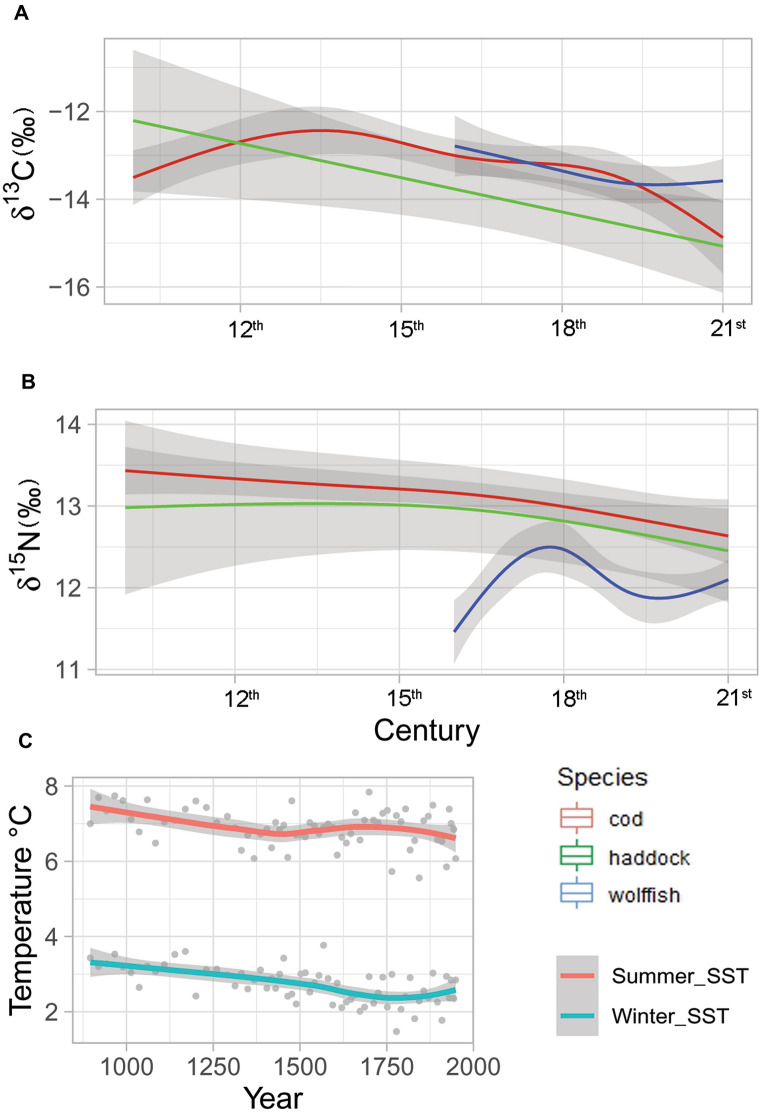
Generalized additive model (GAM) estimates of non-linear pattern of change in δ^13^C (**A**) and δ^15^N (**B**) values over time for Atlantic cod, wolffish and haddock. The bottom panel (**C**) shows the smoothed winter and summer sea surface temperature, as well as temperature estimates (grey dots), based on diatom proxies north of Iceland^27^.

The GAMs that allowed different curves of isotope values for each species across time (Fig. 3) provided a better fit for the data for both δ^13^C and δ^15^N indicating that temporal variation in these values differed between species (AIC 390.00 vs 434.07 for δ^15^N models and AIC 634.15 vs 652.13 for δ^13^C models). The δ^13^C GAM explained 24.1% of the deviance and the δ^15^N GAM explained 40.3%. The model summaries for both δ^13^C and δ^15^N can be found in Table 2.

**Table 2:** Summary of results from the generalized additive models (GAMs).

	δ^13^C				δ^15^N			
	Estimate	SE	t value	p value	Estimate	SE	t value	p value
Intercept	− 13.44	0.11	− 118.70	0.00	13.03	0.06	210.40	0.00

Approximate significance of smooths	edf	Ref. df	F value	p value	edf	Ref. df	F value	p value
Time: cod	4.99	5.52	6.44	0.00	2.61	3.13	5.30	0.00
Time: haddock	1.00	1.00	10.40	0.00	1.98	2.35	2.97	0.05
Time: wolffish	1.00	1.00	0.28	0.59	4.07	4.53	16.49	0.00

**Figure 4:**
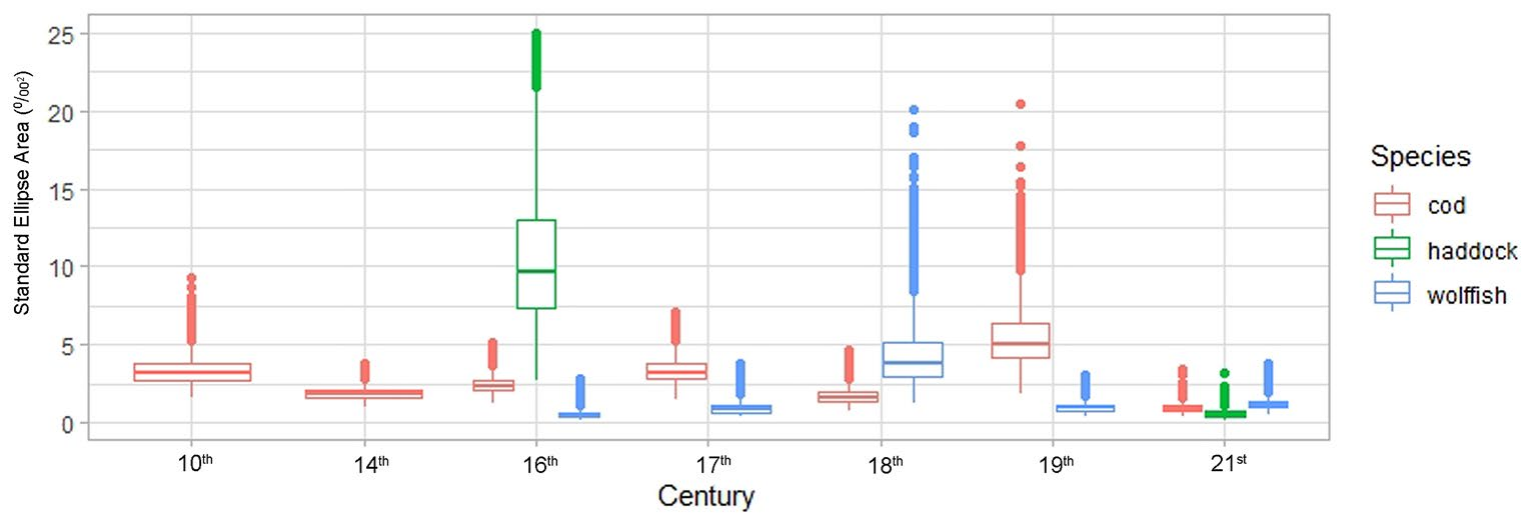
SEA_B_ of demersal fish for each century were a sample size of five or more stable isotope values were available. The boxplots show the distribution of SEA_B_ estimates. Specifically, the median (horizontal black line). First (box) and third (whiskers) quartile. Values falling out with the third quartile are presented as circles.

**Figure 5:**
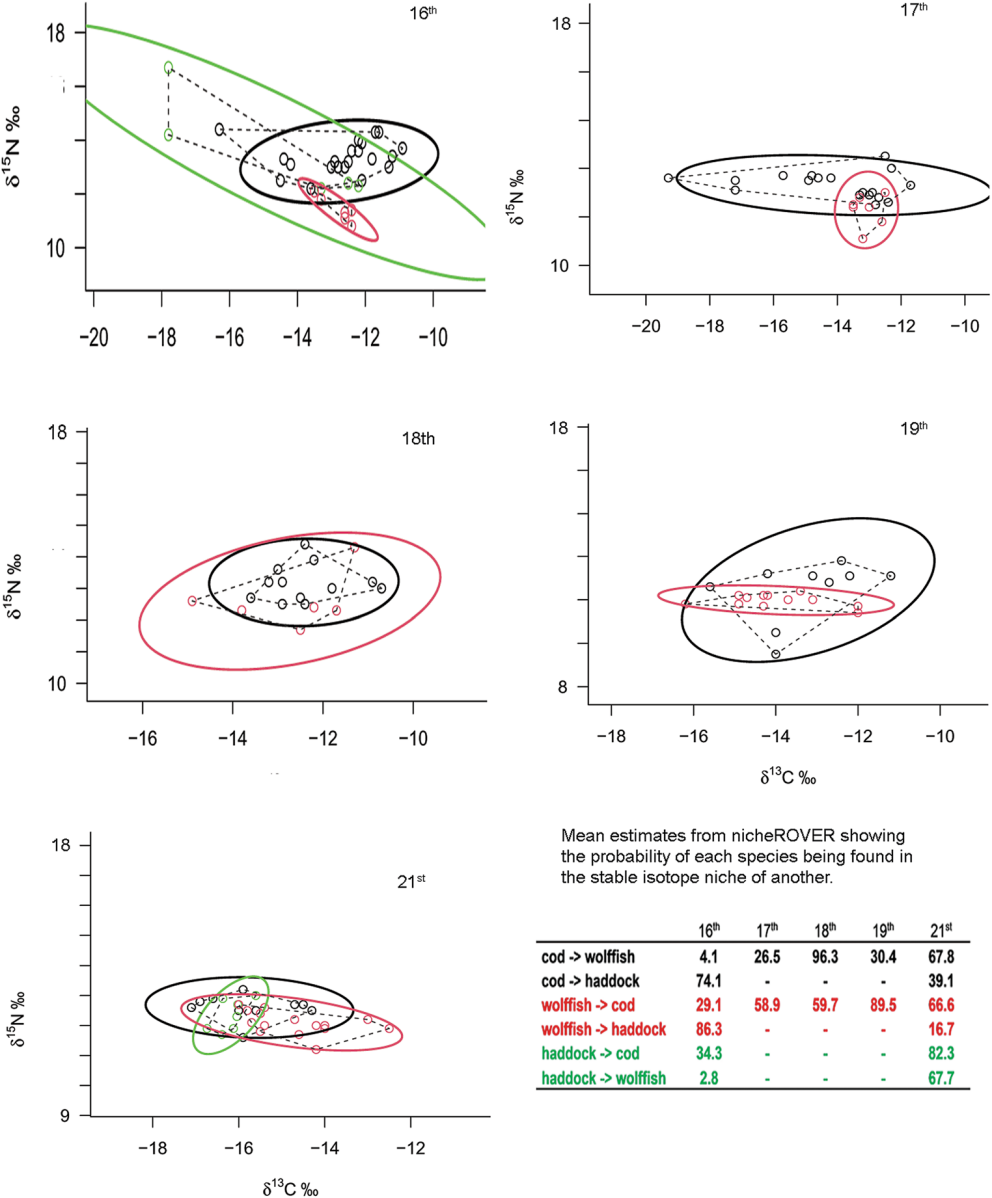
δ^13^C and δ^15^N values and standard ellipses of demersal fish for the centuries were five or more samples of two or more species were available. Atlantic cod and wolffish in the four temporal groups were samples for both species were available. Black ellipses show Atlantic cod, red ellipses are wolffish and green ellipses are haddock. The insert text shows the likelihood of each species being observed in the isotope niche of the others as estimated by nicheROVER (see “Materials and methods”) with the direction of the effect indicated by an arrow.

The stable isotopic niche width (SEA_B_) of Atlantic cod was highest in the tenth, seventeenth and nineteenth centuries and lowest in the present-day sample (Table 1; Fig. 4). The seventeenth and nineteenth centuries and the present-day group differed significantly from all other groups. The stable isotope niche width, SEA_B_, of wolffish was higher in the eighteenth century than at other times (Table 1, Fig. 4). The estimate of niche width of haddock in the sixteenth century was very high but largely explained by two samples. Based on the nicheRover analysis the probability of wolffish being found in the niche of Atlantic cod was lowest in the sixteenth century (19.6%) and highest in the nineteenth century (89.5%). Similarly, the probability of finding Atlantic cod in the isotope niche of wolffish was very low in the sixteenth century (4.1%), highest in the eighteenth century (96.3%) followed by the present-day samples (67.8%) (Fig. 5).

The number of vertebrae growth rings did not correlate to δ^15^N values. See Supplementary Figure S1.

## Discussion

In the current study, analysis of isotope values of zooarchaeological samples from three common species of North Atlantic demersal fish was used to determine variation in fish trophic ecology across several centuries. The results provide a valuable long-term baseline of trophic ecology as they depict both remarkable long-term consistency before modern fisheries, such as the stable trophic level of Atlantic cod, and change of trophic ecology, such as the historically fluctuating trophic level of wolffish. Finally, the data supports present-day convergence of the trophic niches of demersal fish species perhaps driven by intensified fisheries.

Nitrogen values of Atlantic cod bones were constant across all samples before the nineteenth century, when the values dropped, and are lowest in the present-day sample (Fig. 2). A similar drop was observed for haddock but, perhaps because of the low sample size this was not significant. In fact, the estimated smooths by the GAMs suggest that the trophic level of Atlantic cod and haddock has changed in parallel throughout the centuries (Fig. 3). The stable trophic level observed in the early samples is consistent with highly mobile piscivorous fish foraging in an undisturbed ecosystem. The lowered trophic level in more recent times may signal effects of fisheries as it follows the late nineteenth century modernization of the Icelandic fishery when steam trawlers replaced smaller fishing vessels^56^. The advent of trawlers revolutionized cod fishing in Icelandic waters resulting in unprecedented yields^57^.

In contrast to the lowered trophic level of the gadoids there was an increase in the trophic level of wolffish (Figs. 2, 3) and together these patterns largely explain the convergence of stable isotope niches observed in the current study (Fig. 5). Parallel patterns for all species would be expected to result from baseline shifts^40^. However, optimal foraging theory predicts lower trophic levels of species harvested at carrying capacity^58,59^ and harvesting can affect trophic dynamics of top predators directly, as has been shown in sea lions^60^ and fur seals^61^. The current result on the trophic level of Atlantic cod and haddock support a similar effect. Conversely, it is possible that the historical niche of wolffish shifted due to competitive release, bringing the trophic niche of wolffish closer to other demersal fish species. Previous studies have found niche width and niche overlap within communities to change significantly between pristine and modern ecosystems^38,39,62^ and trophic shifts in fish are known to follow competitive release by the reduction of larger or dominant fish species^37,63^. The present-day trophic niche of wolffish is very different from Atlantic cod and it may be more subject to local change in prey availability and disruption to nearshore benthic production as wolffish forage mainly on echinoderms and other benthic invertebrates and less on fish^64^, and this may be reflected in the fluctuating niche observed for wolffish (Figs. 2, 3).

The downward trend of top predator trophic levels, following intensified fishing can also result from fishery induced reduction in the size of individuals as modern fishing has drastically reduced the mean age and size of many fish stocks^65^. Trophic level is known to scale with fish size^66,67^ and a truncated age structure could result in a concurrent decrease in mean δ^15^N values. We could not conclusively test for this effect as our only available proxy of fish size were vertebrae growth rings from Atlantic cod and those were only available for a subset of the total sample (Supplementary Table S2). However, for that subset, the number of vertebrae growth rings did not explain variation in δ^15^N values. In the historical catch most of the individuals represent fully grown fish with a mean age of 9 years or older^31^. Although this is high in comparison with many modern stocks the Icelandic cod stock has been recovering in recent years and for the last 5–10 years the age of the landed cod has been comparable to the estimated age of the archaeological samples, or 8 years and older^68^. Therefore, comparison across centuries may be less confounded by fish age than expected. Moreover, a recent analysis of growth of Atlantic cod otoliths from archaeological excavations in Iceland found that temporal variation in individual growth is slight and most likely to occur at the very early juvenile stage^32^. Historical age and growth information is only available for cod. Nevertheless, we conclude that the noted pattern in lowered δ^15^N values for cod (and haddock) cannot solely be explained by a change in fish size or growth patterns in modern times.

In contrast to the δ^15^N any change in δ^13^C may be more likely to reflect changes in ocean temperature. Seawater temperature is reflected in the δ^13^C values of demersal fish as the carbon signature of primary production is carried through consecutive trophic levels^15^, that is, baseline isotope values will be highly reflected throughout the food web^40^. Described in very broad terms the climate in the North Atlantic started to cool following the Medieval Warm period and was for several centuries characterized by cooler and more fluctuating climate including severe cold spells^26,27^. This period is often collectively referred to as the “Little Ice Age” although the exact timing and climate severity varies within the North Atlantic region^26^. Figure 3 depicts reconstructed winter and summer sea surface temperatures (SST) based on diatom proxies from Northern Iceland^27^ and clearly shows lower temperature, increased variation, including temperature minimums, especially after the seventeenth century. Edvardsson et al.^24^ analyzed carbon values of historical Atlantic cod and SST around Iceland and showed that lower δ^13^C values coincided with cooler periods and periods of more fluctuating climate. This pattern is supported by the current larger dataset and can be visualized from Fig. 3 by the parallel curves of the summer SST and δ^13^C values of cod. No such pattern is noted for either haddock or wolffish although the δ^13^C of both species have a notable downward trend (Fig. 3).

The overall downward trend in carbon values is likely to reflect baseline values in the ocean. However, the lack of species parallels, such as the noted curvature of the δ^13^C values of Atlantic cod and the present day increase in wolffish δ^13^C values may reflect trophic or behavioral effects, such as the historical importance of Atlantic cod migrations. Atlantic cod around Iceland preferentially prey on forage fish and adjust foraging migration to the distribution of capelin^23^, cod will therefore be exposed to a range of sea temperatures and importantly individuals within the stock may experience different lifetime temperature regimes^69^. We suggest that the broad stable isotope niche widths (measured as SEA_B_) observed intermittently for all species during the “Little Ice Age” are also driven by periods of increased temperature variation experiences by individuals within the catch (Fig. 4). In most instances the broader niche is primarily driven by one or a few individuals with very low δ^13^C values. In the case of Atlantic cod this may reflect a more varied origin of the Atlantic cod catch as the broad niche during periods of fluctuating climate contrasts the rather narrow and stable niche width noted for the earliest samples. Climate induced changes in foraging migrations of Atlantic cod are well studied in the twentieth and twenty-first centuries^22,23^ but have likely occurred for much longer, as indicated by otolith shape variation in historical cod catch in Iceland^24^. A mixture of coastal and migratory Atlantic cod in the historical landings would act to increase variability in δ^13^C values as coastal and migratory cod experience very different temperature profiles in their lifetime^61^. A significant exception to carbon driven patterns of broader SEA_B_ is the Atlantic cod high niche width in the nineteenth century that is driven by extremely low δ^15^N values (Figs. 2, 4). This may reflect the period of shift to a lower trophic level as discussed above. Moreover, some of the observed variation in δ^13^C could reflect local variation in primary production or prey species rather than larger patterns of temperature and ocean baseline values. Notably, the upturn of the δ^13^C values of wolffish in present times could suggest that wolffish, in contrast to the gadoid species, are now more dependent on inshore production and this is in fact supported by present day stomach content analysis^62^. If this development continuous it could signal the recurrent divergence of the niches of those demersal fish, following centuries of fishery driven convergence.

To conclude, the current results are consistent with both climate and fishery driven changes. The consistent trophic level of Atlantic cod followed by a convergence of demersal fish δ^15^N values as the lower values of cod and haddock coincide with an increased trophic level of wolffish. Both patterns are in line with predictions based on intensified fishery. In contrast, the parallel downward trend of δ^13^C values likely represent change in baseline values, or ocean temperature, although with two noteworthy exceptions. First, we suggest that the curvature and within group variation of Atlantic cod δ^13^C values reflects life history variation and second, the upturn of δ^13^C values of wolffish in modern times suggest a return to nearshore production. Together, and particularly in the context of the present-day samples, the data provides a valuable baseline of demersal fish historical ecology.

## Supplementary Information

Supplementary Information 1.

Supplementary Information 2.

## Data Availability

The dataset generated and analyzed in the current study is included as Supplementary Table S1.
