# Supplementary material for: A millennium of trophic stability in Atlantic cod (Gadus morhua): transition to a lower and converging trophic niche in modern times
Source: Sci Rep. 2021 Jun 16;11:12681. doi: 10.1038/s41598-021-92243-7 (PMC8209007; doi:10.1038/s41598-021-92243-7)
Supplement: Supplementary file 1 — Supplementary Information 1. [file 41598_2021_92243_MOESM1_ESM.docx]

**A Millennium of trophic stability in Atlantic cod (*Gadus morhua*): transition to a lower and converging trophic niche in modern times.**

**Guðbjörg Ásta Ólafsdóttir^1^*, Ragnar Edvardsson^1^, Sandra Timsic^2^, Ramona Harrison^3^, and William P. Patterson^2^**

^1^ University of Iceland, Research Centre of the Westfjords, Hafnargata 9b, IS415 Bolungarvík, Iceland.

^2^ Saskatchewan Isotope Laboratory, University of Saskatchewan, 114 Science Place, Saskatoon, SK S7N 5E2, Canada.

^3^ University of Bergen, Department of Archaeology, History, Cultural Studies and Religion, Øysteinsgate 3, 5007 Bergen, Norway

[*gaol@hi.is](mailto:*gaol@hi.is)


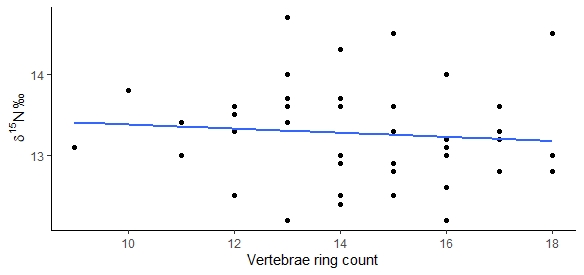


**Supplementary Figure 1.** There was no relationship between δ^15^N values and vertebrae ring count.

**Supplementary Table 2.** Stable isotope values and vertebrae ring counts for the subset of cod vertebrae were this information was available.

ID δ^13^C δ^15^N Count

61596 -13.6 12.2 13

61599 -12.6 13 14

61604 -13 13 11

61610 -12.5 12.4 14

61611 -12.4 13.6 14

61620 -14.5 12.5 12

62513 -13 13.6 12

76430 -14.8 13.7 14

76431 -14.2 13.6 15

76435 -15.7 13.7 13

76436 -17.2 13.5 12

76439 -17.2 13.1 9

77603 -12.7 12.8 17

77604 -11.8 13.3 12

77639 -12.8 12.8 18

77640 -11.7 12.5 14

77641 -11.8 14.7 13

77642 -13 12.6 16

77643 -11.7 14.3 14

77644 -14.5 13.8 10

77645 -12.1 12.9 14

77646 -12.7 13.3 17

77649 -11.6 12.2 16

77650 -12.2 14 16

77651 -11.2 13.6 13

77652 -10.7 13.4 11

77653 -11.4 13.4 13

77654 -12.8 13 16

77656 -12.5 14.5 15

77659 -15.3 14.5 18

77662 -13.3 14 13

77663 -12.2 13.6 17

77665 -13.1 13.1 16

77666 -12.6 12.5 15

78476 -12.9 13.2 16

78477 -11.7 13.3 15

78478 -19.3 13.6 14

78479 -14 12.5 15

78480 -15.9 12.9 15

78481 -17.6 13.2 17

78482 -12.7 12.8 15

78483 -12.6 13 18
