# Supplementary material for: A millennium of trophic stability in Atlantic cod (Gadus morhua): transition to a lower and converging trophic niche in modern times
Source: Sci Rep. 2021 Jun 16;11:12681. doi: 10.1038/s41598-021-92243-7 (PMC8209007; doi:10.1038/s41598-021-92243-7)
Supplement: Supplementary file 2 — Supplementary Information 2. [file 41598_2021_92243_MOESM2_ESM.docx]

**A Millennium of trophic stability in Atlantic cod (*Gadus morhua*): transition to a lower and converging trophic niche in modern times.**

**Guðbjörg Ásta Ólafsdóttir^1^*, Ragnar Edvardsson^1^, Sandra Timsic^2^, Ramona Harrison^3^, and William P. Patterson^2^**

^1^ University of Iceland, Research Centre of the Westfjords, Hafnargata 9b, IS415 Bolungarvík, Iceland.

^2^ Saskatchewan Isotope Laboratory, University of Saskatchewan, 114 Science Place, Saskatoon, SK S7N 5E2, Canada.

^3^ University of Bergen, Department of Archaeology, History, Cultural Studies and Religion, Øysteinsgate 3, 5007 Bergen, Norway

[*gaol@hi.is](mailto:*gaol@hi.is)

**Supplementary Table S1.** The table provides information on the δ^13^C, δ^15^N, %C and %N values of each fish sample used in the analysis. Please refer to the main text for further information.

| ID | Site | Excavation | Context | Species | %C | C | %N | N | C/N ratio | Century | **Dating** |
| --- | --- | --- | --- | --- | --- | --- | --- | --- | --- | --- | --- |
| 61433 | BRV | 12 | 3 | haddock | 48.4 | -15.2 | 14.5 | 13.1 | 3.34 | 18 | ^14^C (1554±30) |
| 61596 | BRV | 12 | 5 | cod | 45.6 | -13.6 | 15.7 | 12.2 | 3.4 | 16 | ^14^C (1554±30) |
| 61598 | BRV | 12 | 5 | cod | 36.3 | -12.5 | 11.3 | 13.2 | 3.23 | 16 | ^14^C (1554±30) |
| 61599 | BRV | 12 | 5 | cod | 41.3 | -12.6 | 13.7 | 13 | 3.51 | 16 | ^14^C (1554±30) |
| 61604 | BRV | 12 | 5 | cod | 45.5 | -13 | 15.2 | 13 | 3.5 | 16 | ^14^C (1554±30) |
| 61606 | BRV | 12 | 5 | cod | 9.1 | -16.3 | 2.6 | 14.4 | 3.51 | 16 | ^14^C (1554±30) |
| 61610 | BRV | 12 | 5 | cod | 47 | -12.5 | 16.3 | 12.4 | 3.37 | 16 | ^14^C (1554±30) |
| 61611 | BRV | 12 | 5 | cod | 44.7 | -12.4 | 15.5 | 13.6 | 3.37 | 16 | ^14^C (1554±30) |
| 61615 | BRV | 12 | 5 | cod | 33.7 | -14.2 | 10.3 | 13.1 | 3.26 | 16 | ^14^C (1554±30) |
| 61618 | BRV | 12 | 5 | cod | 38.7 | -14.4 | 12.2 | 13.3 | 3.18 | 16 | ^14^C (1554±30) |
| 61619 | BRV | 12 | 5 | cod | 41.2 | -12.9 | 13.1 | 13.2 | 3.15 | 16 | ^14^C (1554±30) |
| 61620 | BRV | 12 | 5 | cod | 45.1 | -14.5 | 15.4 | 12.5 | 3.44 | 16 | ^14^C (1554±30) |
| 61656 | BRV | 12 | 5 | haddock | 7.2 | -17.8 | 3.1 | 14.2 | 2.31 | 16 | ^14^C (1554±30) |
| 61659 | BRV | 12 | 5 | haddock | 11.6 | -17.8 | 3.7 | 15.7 | 3.18 | 16 | ^14^C (1554±30) |
| 62513 | BRV | 12 | 3 | cod | 42.5 | -13 | 14.5 | 13.6 | 2.94 | 18 | ^14^C (1795±28) |
| 63827 | BRV | 12 | 3 | cod | 49.4 | -10.9 | 17 | 13.2 | 3.41 | 18 | ^14^C (1795±28) |
| 63832 | BRV | 12 | 3 | cod | 43.9 | -12.2 | 15.4 | 13.9 | 3.34 | 18 | ^14^C (1795±28) |
| 63845 | BRV | 12 | 3 | cod | 49.4 | -10.7 | 17.4 | 13 | 3.32 | 18 | ^14^C (1795±28) |
| 63903 | BRV | 12 | 4 | cod | 46.1 | -13.6 | 15.5 | 12.7 | 3.47 | 18 | ^14^C (1795±28) |
| 76430 | BRV | 15 | 7 | cod | 20.4 | -14.8 | 7.8 | 13.7 | 3.07 | 17 | Context (1637-1680) |
| 76431 | BRV | 15 | 7 | cod | 20.6 | -14.2 | 7.8 | 13.6 | 3.07 | 17 | Context (1637-1680) |
| 76435 | BRV | 15 | 7 | cod | 14.8 | -15.7 | 5.6 | 13.7 | 3.07 | 17 | Context (1637-1680) |
| 76436 | BRV | 15 | 7 | cod | 12.9 | -17.2 | 5 | 13.5 | 3.06 | 17 | Context (1637-1680) |
| 76439 | BRV | 15 | 7 | cod | 16.6 | -17.2 | 6.4 | 13.1 | 3.05 | 17 | Context (1637-1680) |
| 77603 | BRV | 15 | 12 | cod | 14.6 | -12.7 | 5 | 12.8 | 3.38 | 19 | ^14^C (1890±32) |
| 77604 | BRV | 15 | 14 | cod | 18.2 | -11.8 | 6.5 | 13.3 | 3.27 | 16 | Context (1410-1649) |
| 77639 | BRV | 15 | 8 | cod | 15.7 | -12.8 | 5.8 | 12.8 | 3.14 | 10 | ^14^C (970±30) |
| 77640 | BRV | 15 | 16 | cod | 15.2 | -11.7 | 5.8 | 12.5 | 3.07 | 14 | ^14^C (1410±30) |
| 77641 | BRV | 15 | 8 | cod | 15.1 | -11.8 | 5.7 | 14.7 | 3.1 | 10 | ^14^C (970±30) |
| 77642 | BRV | 15 | 17 | cod | 15.6 | -13 | 5.8 | 12.6 | 3.12 | 14 | Context (≤ 1410) |
| 77643 | BRV | 15 | 14 | cod | 21.3 | -11.7 | 7.9 | 14.3 | 3.15 | 16 | Context (1410-1649) |
| 77644 | BRV | 15 | 8 | cod | 7.3 | -14.5 | 2.7 | 13.8 | 3.18 | 10 | ^14^C (970±30) |
| 77645 | BRV | 15 | 16 | cod | 10.9 | -12.1 | 4 | 12.9 | 3.22 | 14 | ^14^C (1410±30) |
| 77646 | BRV | 15 | 16 | cod | 16.5 | -12.7 | 6.1 | 13.3 | 3.16 | 14 | ^14^C (1410±30) |
| 77649 | BRV | 15 | 16 | cod | 14 | -11.6 | 5.1 | 12.2 | 3.19 | 14 | ^14^C (1410±30) |
| 77650 | BRV | 15 | 14 | cod | 19.7 | -12.2 | 7.2 | 14 | 3.2 | 16 | Context (1410-1649) |
| 77651 | BRV | 15 | 8 | cod | 11.1 | -11.2 | 4 | 13.6 | 3.25 | 10 | ^14^C (970±30) |
| 77652 | BRV | 15 | 16 | cod | 12.6 | -10.7 | 4.6 | 13.4 | 3.18 | 14 | ^14^C (1410±30) |
| 77653 | BRV | 15 | 17 | cod | 18.4 | -11.4 | 6.7 | 13.4 | 3.24 | 14 | Context (≤ 1410) |
| 77654 | BRV | 15 | 14 | cod | 18.6 | -12.8 | 6.7 | 13 | 3.25 | 16 | Context (1410-1649) |
| 77656 | BRV | 15 | 7 | cod | 10.2 | -12.5 | 3.6 | 14.5 | 3.36 | 17 | Context (1637-1680) |
| 77659 | BRV | 15 | 8 | cod | 15.4 | -15.3 | 5.5 | 14.5 | 3.29 | 10 | ^14^C (970±30) |
| 77662 | BRV | 15 | 8 | cod | 9.3 | -13.3 | 3.3 | 14 | 3.25 | 10 | ^14^C (970±30) |
| 77663 | BRV | 15 | 14 | cod | 23.5 | -12.2 | 8.7 | 13.6 | 3.18 | 16 | Context (1410-1649) |
| 77665 | BRV | 15 | 12 | cod | 17.6 | -13.1 | 6.2 | 13.1 | 3.34 | 19 | ^14^C (1890±32) |
| 77666 | BRV | 15 | 17 | cod | 26.4 | -12.6 | 10 | 12.5 | 3.1 | 14 | Context (≤ 1410) |
| 78476 | BRV | 15 | 3 | cod | 13.6 | -12.9 | 4.9 | 13.2 | 3.21 | 18 | ^14^C (1820±32) |
| 78477 | BRV | 15 | 7 | cod | 19.9 | -11.7 | 7.4 | 13.3 | 3.16 | 17 | Context (1637-1680) |
| 78478 | BRV | 15 | 7 | cod | 7.1 | -19.3 | 2.4 | 13.6 | 3.44 | 17 | Context (1637-1680) |
| 78479 | BRV | 15 | 8 | cod | 14.6 | -14 | 5.3 | 12.5 | 3.24 | 10 | ^14^C (970±30) |
| 78480 | BRV | 15 | 8 | cod | 14.7 | -15.9 | 5.5 | 12.9 | 3.14 | 10 | ^14^C (970±30) |
| 78481 | BRV | 15 | 8 | cod | 10.5 | -17.6 | 4 | 13.2 | 3.07 | 10 | ^14^C (970±30) |
| 78482 | BRV | 15 | 12 | cod | 19 | -12.7 | 7 | 12.8 | 3.18 | 19 | ^14^C (1890±32) |
| 78483 | BRV | 15 | 16 | cod | 16.2 | -12.6 | 6 | 13 | 3.16 | 14 | ^14^C (1410±30) |
| 79848 | BRV | 15 | 1 | wolffish | 33.4 | -14.7 | 11.1 | 12.1 | 3.53 | 19 | Context (>1890) |
| 79850 | BRV | 15 | 1 | wolffish | 32.9 | -16.2 | 10 | 11.8 | 3.83 | 19 | Context (>1890) |
| 79857 | BRV | 15 | 3 | wolffish | 30.7 | -14.9 | 10.1 | 12.6 | 3.56 | 18 | ^14^C (1820±32) |
| 79858 | BRV | 15 | 3 | wolffish | 24.6 | -13.8 | 8.6 | 12.3 | 3.35 | 18 | ^14^C (1820±32) |
| 79859 | BRV | 15 | 3 | cod | 22.7 | -12.5 | 8.1 | 12.7 | 3.27 | 18 | ^14^C (1820±32) |
| 79860 | BRV | 15 | 3 | cod | 23.3 | -12.4 | 8.5 | 12.5 | 3.21 | 18 | ^14^C (1820±32) |
| 79864 | BRV | 15 | 6 | wolffish | 32.2 | -13.2 | 12.2 | 11.1 | 3.09 | 17 | Context (1637-1680) |
| 79865 | BRV | 15 | 6 | cod | 25.9 | -12.4 | 9.8 | 12.6 | 3.11 | 17 | Context (1637-1680) |
| 79868 | BRV | 15 | 6 | cod | 20.6 | -12.8 | 7.6 | 12.5 | 3.18 | 17 | Context (1637-1680) |
| 79869 | BRV | 15 | 6 | cod | 19.7 | -13.2 | 7.1 | 13 | 3.25 | 17 | Context (1637-1680) |
| 79870 | BRV | 15 | 6 | cod | 30.9 | -14.9 | 11.4 | 13.5 | 3.17 | 17 | Context (1637-1680) |
| 79871 | BRV | 15 | 6 | cod | 21.4 | -13 | 7.9 | 12.9 | 3.17 | 17 | Context (1637-1680) |
| 79872 | BRV | 15 | 6 | cod | 18.3 | -13.2 | 6.6 | 13 | 3.23 | 17 | Context (1637-1680) |
| 79873 | BRV | 15 | 6 | cod | 29.6 | -14.6 | 11.1 | 13.6 | 3.12 | 17 | Context (1637-1680) |
| 79874 | BRV | 15 | 6 | cod | 21.4 | -12.7 | 8 | 12.8 | 3.12 | 17 | Context (1637-1680) |
| 79875 | BRV | 15 | 6 | cod | 20.2 | -12.3 | 7.4 | 14 | 3.17 | 17 | Context (1637-1680) |
| 79876 | BRV | 15 | 6 | cod | 11.1 | -13.3 | 3.9 | 12.9 | 3.29 | 17 | Context (1637-1680) |
| 79877 | BRV | 15 | 6 | cod | 19.5 | -12.9 | 7.4 | 13 | 3.07 | 17 | Context (1637-1680) |
| 79878 | BRV | 15 | 8 | haddock | 23.8 | -11.3 | 8.9 | 13.6 | 3.11 | 10 | ^14^C (970±30) |
| 80625 | BRV | 15 | 8 | haddock | 17.6 | -12.8 | 6.4 | 11.9 | 3.23 | 10 | ^14^C (970±30) |
| 80627 | BRV | 15 | 8 | cod | 30.4 | -14.9 | 10.5 | 13.1 | 3.38 | 10 | ^14^C (970±30) |
| 80628 | BRV | 15 | 8 | cod | 30.3 | -13.9 | 11.1 | 12.6 | 3.18 | 10 | ^14^C (970±30) |
| 80629 | BRV | 15 | 8 | cod | 26 | -14 | 9.1 | 13.6 | 3.32 | 10 | ^14^C (970±30) |
| 80630 | BRV | 15 | 8 | cod | 31.5 | -13 | 11.4 | 13.6 | 3.25 | 10 | ^14^C (970±30) |
| 80631 | BRV | 15 | 8 | cod | 31.6 | -12.4 | 11.1 | 13.4 | 3.33 | 10 | ^14^C (970±30) |
| 80632 | BRV | 15 | 8 | cod | 28.2 | -12.9 | 10.1 | 14.4 | 3.28 | 10 | ^14^C (970±30) |
| 80635 | BRV | 15 | 8 | cod | 28.8 | -11.7 | 10.7 | 13.4 | 3.17 | 10 | ^14^C (970±30) |
| 80636 | BRV | 15 | 8 | cod | 32.2 | -12.4 | 11.7 | 13.6 | 3.22 | 10 | ^14^C (970±30) |
| 80637 | BRV | 15 | 8 | cod | 29.6 | -12.9 | 10.9 | 12.8 | 3.17 | 10 | ^14^C (970±30) |
| 80638 | BRV | 15 | 8 | cod | 30.4 | -12.7 | 11.1 | 13 | 3.2 | 10 | ^14^C (970±30) |
| 80639 | BRV | 15 | 8 | cod | 14.1 | -12.2 | 5.2 | 13.1 | 3.2 | 19 | ^14^C (1890±32) |
| 80640 | BRV | 15 | 12 | wolffish | 12 | -14.9 | 3.9 | 12.2 | 3.56 | 19 | ^14^C (1890±32) |
| 80641 | BRV | 15 | 12 | wolffish | 11.4 | -14.3 | 3.8 | 12.2 | 3.49 | 19 | ^14^C (1890±32) |
| 80642 | BRV | 15 | 8 | cod | 12.4 | -14.2 | 4 | 13.2 | 3.58 | 19 | ^14^C (1890±32) |
| 80647 | BRV | 15 | 14 | haddock | 13.2 | -12.5 | 4.5 | 12.4 | 3.45 | 16 | Context (1410-1649) |
| 80648 | BRV | 15 | 14 | wolffish | 11.8 | -12.6 | 4.2 | 11.3 | 3.33 | 16 | Context (1410-1649) |
| 80649 | BRV | 15 | 14 | wolffish | 26.9 | -12.4 | 9.9 | 11.4 | 3.19 | 16 | Context (1410-1649) |
| 80650 | BRV | 15 | 14 | wolffish | 13.6 | -12.6 | 4.8 | 11.1 | 3.34 | 16 | Context (1410-1649) |
| 80905 | BRV | 15 | 16 | haddock | 36.9 | -12.2 | 14.1 | 12.8 | 3.06 | 14 | ^14^C (1410±30) |
| 80906 | BRV | 15 | 16 | haddock | 37.8 | -12.3 | 14.5 | 12.8 | 3.05 | 14 | ^14^C (1410±30) |
| 80918 | BRV | 15 | 16 | cod | 30.6 | -12.7 | 11.6 | 12.1 | 3.09 | 14 | ^14^C (1410±30) |
| 80919 | BRV | 15 | 16 | cod | 30.8 | -11.2 | 11.9 | 13.9 | 3.03 | 14 | ^14^C (1410±30) |
| 80920 | BRV | 15 | 16 | cod | 30.2 | -12.3 | 11.5 | 12.6 | 3.06 | 14 | ^14^C (1410±30) |
| 80921 | BRV | 15 | 16 | cod | 34.2 | -11.8 | 12.9 | 14 | 3.11 | 14 | ^14^C (1410±30) |
| 80922 | BRV | 15 | 16 | cod | 19.5 | -12 | 7.1 | 13.8 | 3.2 | 14 | ^14^C (1410±30) |
| 80923 | BRV | 15 | 16 | cod | 29.3 | -12.5 | 11 | 13.2 | 3.12 | 14 | ^14^C (1410±30) |
| 80924 | BRV | 15 | 16 | cod | 35.9 | -12.1 | 13.7 | 12.7 | 3.07 | 14 | ^14^C (1410±30) |
| 80925 | BRV | 15 | 16 | cod | 34.4 | -13 | 13.1 | 12.9 | 3.08 | 14 | ^14^C (1410±30) |
| 80926 | BRV | 15 | 16 | cod | 26.3 | -12.1 | 10.1 | 12.1 | 3.06 | 14 | ^14^C (1410±30) |
| 80929 | SGN | NA | 102 | cod | 30.1 | -12.5 | 11.1 | 12.7 | 3.19 | 14 | Context (< 1239) |
| 80931 | SGN | NA | 101 | cod | 28.7 | -12.8 | 10.5 | 13.9 | 3.22 | 14 | ^14^C (1239±25) |
| 80932 | SGN | NA | 187 | cod | 37.7 | -13 | 14 | 14.4 | 3.16 | 14 | ^14^C (1350±50) |
| 81117 | BRV | 15 | 12 | haddock | 21.7 | -13.4 | 7.4 | 12.4 | 3.44 | 19 | ^14^C (1890±32) |
| 81129 | BRV | 15 | 16 | cod | 33.1 | -11.8 | 12.5 | 13.6 | 3.11 | 14 | ^14^C (1410±30) |
| 81130 | SGN | NA | 102 | cod | 39.6 | -14.5 | 14.2 | 13.8 | 3.26 | 14 | Context (< 1239) |
| 81131 | SGN | NA | 187 | cod | 20.1 | -14.7 | 7.2 | 12.8 | 3.28 | 14 | ^14^C (1350±50) |
| 81133 | SGN | NA | 187 | cod | 19 | -13 | 7 | 13.4 | 3.15 | 14 | ^14^C (1350±50) |
| 83519 | ISA | NA | NA | haddock | 43.1 | -14.83394 | 12.6 | 11.9 | 3.43 | 21 | 2018 |
| 83520 | ISA | NA | NA | haddock | 42.2 | -15.10151 | 12.2 | 11.7 | 3.47 | 21 | 2018 |
| 83521 | ISA | NA | NA | haddock | 43.8 | -15.08075 | 12.6 | 12.9 | 3.47 | 21 | 2018 |
| 83524 | ISA | NA | NA | haddock | 44.4 | -15.44631 | 12.4 | 11.9 | 3.59 | 21 | 2018 |
| 83526 | ISA | NA | NA | haddock | 44 | -14.73328 | 12.4 | 12.7 | 3.55 | 21 | 2018 |
| 83527 | ISA | NA | NA | haddock | 43.6 | -14.75384 | 12.8 | 12.3 | 3.39 | 21 | 2018 |
| 83528 | ISA | NA | NA | haddock | 43.2 | -14.31259 | 12.9 | 13 | 3.36 | 21 | 2018 |
| 83532 | ISA | NA | NA | cod | 36.1 | -14.3 | 14 | 12.5 | 3.02 | 21 | 2018 |
| 83533 | ISA | NA | NA | cod | 35.3 | -14.7 | 13.6 | 12.7 | 3.03 | 21 | 2018 |
| 83536 | ISA | NA | NA | cod | 33.6 | -14.5 | 13 | 12.7 | 3.03 | 21 | 2018 |
| 83539 | ISA | NA | NA | cod | 34.7 | -15.6 | 13.5 | 12.5 | 3.02 | 21 | 2018 |
| 83540 | ISA | NA | NA | cod | 38.9 | -16 | 14.8 | 12.5 | 3.06 | 21 | 2018 |
| 85064 | BRV | 19 | 2 | wolffish | 37.1 | -14.9 | 12.3 | 11.8 | 3.53 | 19 | Context (>1890) |
| 85065 | BRV | 19 | 2 | wolffish | 37.4 | -14.2 | 12.5 | 12.2 | 3.51 | 19 | Context (>1890) |
| 85066 | BRV | 19 | 2 | wolffish | 40.5 | -14.3 | 13.4 | 11.7 | 3.53 | 19 | Context (>1890) |
| 85067 | BRV | 19 | 2 | wolffish | 36.3 | -14.2 | 12.1 | 12.2 | 3.5 | 19 | Context (>1890) |
| 85068 | BRV | 19 | 4 | cod | 33.7 | -14 | 11.6 | 10.5 | 3.39 | 19 | ^14^C (1860±27) |
| 85069 | BRV | 19 | 4 | cod | 34.6 | -14 | 11.6 | 9.5 | 3.5 | 19 | ^14^C (1860±27) |
| 85070 | BRV | 19 | 7 | cod | 27.5 | -12.4 | 10.1 | 14.4 | 3.17 | 18 | ^14^C (1770±27) |
| 85071 | BRV | 19 | 7 | cod | 32.1 | -12.9 | 11.9 | 12.5 | 3.16 | 18 | ^14^C (1770±27) |
| 85074 | BRV | 19 | 7 | cod | 29.1 | -13.2 | 10.8 | 13.2 | 3.16 | 18 | ^14^C (1770±27) |
| 85075 | BRV | 19 | 7 | cod | 30.5 | -11.8 | 11 | 13 | 3.25 | 18 | ^14^C (1770±27) |
| 85076 | BRV | 19 | 7 | wolffish | 39.7 | -11.7 | 14.5 | 12.3 | 3.2 | 18 | ^14^C (1788±27) |
| 85077 | BRV | 19 | 7 | wolffish | 38.5 | -11.3 | 14.6 | 14.3 | 3.09 | 18 | ^14^C (1788±27) |
| 85078 | BRV | 19 | 8 | wolffish | 36.4 | -12.2 | 13.4 | 12.4 | 3.17 | 18 | ^14^C (1788±27) |
| 85079 | BRV | 19 | 8 | wolffish | 39.7 | -12.5 | 14.9 | 11.7 | 3.12 | 18 | ^14^C (1788±27) |
| 85080 | BRV | 19 | 13 | cod | 37.3 | -11.2 | 13.9 | 13.1 | 3.13 | 19 | ^14^C (1880±27) |
| 85081 | BRV | 19 | 13 | cod | 31.3 | -15.6 | 11.1 | 12.6 | 3.3 | 19 | ^14^C (1880±27) |
| 85082 | BRV | 19 | 13 | cod | 32.9 | -12.4 | 11.8 | 13.8 | 3.26 | 19 | ^14^C (1880±27) |
| 85083 | BRV | 19 | 13 | wolffish | 29.7 | -13.1 | 10.6 | 12 | 3.27 | 19 | ^14^C (1880±27) |
| 85086 | BRV | 19 | 13 | wolffish | 38.1 | -12 | 14.3 | 11.4 | 3.11 | 19 | ^14^C (1880±27) |
| 85087 | BRV | 19 | 13 | wolffish | 36.2 | -12 | 13.7 | 11.7 | 3.09 | 19 | ^14^C (1880±27) |
| 85108 | BRV | 19 | 14 | wolffish | 34.3 | -12.5 | 12.2 | 13 | 3.28 | 17 | Context (~1650) |
| 85109 | BRV | 19 | 14 | wolffish | 36.5 | -12.6 | 13.6 | 11.8 | 3.14 | 17 | Context (~1650) |
| 85110 | BRV | 19 | 14 | wolffish | 30.1 | -13.5 | 10.1 | 12.5 | 3.48 | 17 | Context (~1650) |
| 85111 | BRV | 19 | 14 | wolffish | 28.3 | -13 | 9.8 | 12.4 | 3.37 | 17 | Context (~1650) |
| 85112 | BRV | 19 | 14 | wolffish | 33.9 | -13.3 | 11.7 | 12.8 | 3.39 | 17 | Context (~1650) |
| 85113 | BRV | 19 | 14 | wolffish | 30.9 | -13.5 | 10.8 | 12.4 | 3.34 | 17 | Context (~1650) |
| 85114 | BRV | 19 | 15 | cod | 35.5 | -11.6 | 13.2 | 14.3 | 3.14 | 16 | Context (1410-1649) |
| 85115 | BRV | 19 | 15 | cod | 33.6 | -10.9 | 12.5 | 13.7 | 3.13 | 16 | Context (1410-1649) |
| 85117 | BRV | 19 | 15 | cod | 27.1 | -11.3 | 10 | 13 | 3.18 | 16 | Context (1410-1649) |
| 85120 | BRV | 19 | 15 | cod | 35.7 | -12.1 | 13.2 | 13.9 | 3.16 | 16 | Context (1410-1649) |
| 85121 | BRV | 19 | 15 | cod | 35.3 | -11.2 | 13.2 | 13.4 | 3.13 | 16 | Context (1410-1649) |
| 85122 | BRV | 19 | 15 | cod | 34.2 | -12.1 | 12.8 | 12.5 | 3.12 | 16 | Context (1410-1649) |
| 85123 | BRV | 19 | 15 | wolffish | 27.9 | -13.5 | 10.1 | 12.1 | 3.23 | 16 | Context (1410-1649) |
| 85124 | BRV | 19 | 15 | wolffish | 27.6 | -12.4 | 10.4 | 10.8 | 3.11 | 16 | Context (1410-1649) |
| 85126 | BRV | 19 | 15 | wolffish | 39.9 | -13.3 | 14.3 | 11.8 | 3.27 | 16 | Context (1410-1649) |
| 85127 | BRV | 19 | 15 | haddock | 29.6 | -12.2 | 11 | 12.3 | 3.15 | 16 | Context (1410-1649) |
| 85128 | BRV | 19 | 15 | haddock | 32.6 | -13.3 | 12.2 | 12.2 | 3.13 | 16 | Context (1410-1649) |
| 85133 | BRV | 19 | 13 | wolffish | 28.6 | -13.7 | 10.3 | 12 | 3.26 | 19 | ^14^C (1880±27) |
| 86037 | ISA | NA | NA | wolffish | 42.3 | -14.21 | 13.6 | 11.8 | 3.12 | 21 | 2021 |
| 86038 | ISA | NA | NA | wolffish | 42.1 | -12.71 | 15.5 | 11.9 | 2.71 | 21 | 2021 |
| 86040 | ISA | NA | NA | wolffish | 42 | -12.71 | 15.3 | 12 | 2.75 | 21 | 2021 |
| 86041 | ISA | NA | NA | wolffish | 41.5 | -11.71 | 14.8 | 12.2 | 2.79 | 21 | 2021 |
| 86042 | ISA | NA | NA | wolffish | 42.3 | -12.91 | 15.2 | 11.2 | 2.78 | 21 | 2021 |
| 86043 | ISA | NA | NA | wolffish | 41.8 | -13.41 | 13.8 | 12.2 | 3.03 | 21 | 2021 |
| 86044 | ISA | NA | NA | wolffish | 40.2 | -14.51 | 12.7 | 12.5 | 3.16 | 21 | 2021 |
| 86045 | ISA | NA | NA | wolffish | 42.7 | -14.51 | 13.1 | 12.5 | 3.26 | 21 | 2021 |
| 86046 | ISA | NA | NA | wolffish | 42.3 | -14.71 | 12.7 | 12.7 | 3.33 | 21 | 2021 |
| 86067 | ISA | NA | NA | wolffish | 45.3 | -12.91 | 15.3 | 12 | 2.95 | 21 | 2021 |
| 86070 | ISA | NA | NA | wolffish | 42.5 | -14.21 | 13.2 | 12.4 | 3.22 | 21 | 2021 |
| 86071 | ISA | NA | NA | wolffish | 42.7 | -14.11 | 13.5 | 12 | 3.16 | 21 | 2021 |
| 86072 | ISA | NA | NA | wolffish | 43.3 | -11.21 | 16.2 | 11.9 | 2.68 | 21 | 2021 |
| 86073 | ISA | NA | NA | wolffish | 43.1 | -13.31 | 14.4 | 11.7 | 3 | 21 | 2021 |
| 86074 | ISA | NA | NA | wolffish | 44.9 | -14.11 | 13.7 | 12.6 | 3.28 | 21 | 2021 |
| 86075 | ISA | NA | NA | wolffish | 43.8 | -14.41 | 13.4 | 12.1 | 3.27 | 21 | 2021 |
| 86078 | ISA | NA | NA | cod | 42.3 | -15.81 | 12.4 | 12.6 | 3.42 | 21 | 2021 |
| 86079 | ISA | NA | NA | cod | 44.4 | -15.61 | 12.8 | 12.8 | 3.46 | 21 | 2021 |
| 86080 | ISA | NA | NA | cod | 42.7 | -14.61 | 13 | 13.2 | 3.29 | 21 | 2021 |
| 86087 | ISA | NA | NA | cod | 42.6 | -14.61 | 14.6 | 11.6 | 2.93 | 21 | 2021 |
| 86088 | ISA | NA | NA | cod | 43.8 | -15.31 | 14 | 12.9 | 3.13 | 21 | 2021 |
